# The genome sequence of the Barred Straw, *Gandaritis pyraliata *(Denis & Schiffermüller, 1775)

**DOI:** 10.12688/wellcomeopenres.19526.1

**Published:** 2023-10-10

**Authors:** Douglas Boyes, Peter W.H. Holland, University of Oxford and Wytham Woods Genome Acquisition Lab, Darwin Tree of Life Barcoding collective, Wellcome Sanger Institute Tree of Life programme, Wellcome Sanger Institute Scientific Operations: DNA Pipelines collective, Tree of Life Core Informatics collective, Darwin Tree of Life Consortium

**Affiliations:** 1 UK Centre for Ecology & Hydrology, Wallingford, England, UK; 2 University of Oxford, Oxford, England, UK

**Keywords:** Gandaritis pyraliata, the Barred Straw, genome sequence, chromosomal, Lepidoptera

## Abstract

We present a genome assembly from an individual female *Gandaritis pyraliata* (the Barred Straw; Arthropoda; Insecta; Lepidoptera; Geometridae). The genome sequence is 295.6 megabases in span. Most of the assembly is scaffolded into 31 chromosomal pseudomolecules, including the W and Z sex chromosomes. The mitochondrial genome has also been assembled and is 15.74 kilobases in length. Gene annotation of this assembly on Ensembl identified 15,805 protein coding genes.

## Species taxonomy

Eukaryota; Metazoa; Eumetazoa; Bilateria; Protostomia; Ecdysozoa; Panarthropoda; Arthropoda; Mandibulata; Pancrustacea; Hexapoda; Insecta; Dicondylia; Pterygota; Neoptera; Endopterygota; Amphiesmenoptera; Lepidoptera; Glossata; Neolepidoptera; Heteroneura; Ditrysia; Obtectomera; Geometroidea; Geometridae; Larentiinae; *Gandaritis*; *Gandaritis pyraliata* (Denis & Schiffermüller, 1775) (NCBI:txid934938).

## Background

The resting position of an insect can be as important as shape and colour for effective camouflage or mimicry. Most moths of the family Geometridae rest with their wings flat against the substrate, often enabling wing colour and pattern to blend with surroundings. The Barred Straw *Gandaritis pyraliata* (synonym *Eulithis pyraliata*) is an exception. *G. pyraliata* invariably rests with its wings outstretched and held aloft at an angle above horizontal; the hindwings are also rotated forward under the forewings, with the trailing edge curled. Only one pair of wings is visible, therefore, stretched out and partially raised. The adaptive significance of this unusual resting position is unclear. One possibility is that when settled in herbaceous vegetation, rather than on tree trunks, the shape may appear less ‘moth-like’ to visual predators.

As the common name suggests, *Gandaritis pyraliata* is a pale straw-coloured moth with a jagged, black-edged band across each forewing. The species is widely distributed across Europe and Asia, and in Britain it is found as far north as Orkney ( GBIF Secretariat, 2022; Randle *et al.*, 2019). The larvae feed primarily on *Galium aparine* (cleavers), a plant avoided by many insects due to the presence of deterrent chemicals including alkaloids, glycosides and phenolic compounds ( Morimoto *et al.,* 2005). In southern Britain, the adult moth is on the wing from June to August, with eggs overwintering and larvae developing from April to June ( South, 1961; Waring *et al.,* 2017).

The complete genome sequence of *Gandaritis pyraliata* was determined as part of the Darwin Tree of Life project. The assembled genome will facilitate research into the evolutionary arms race between insect and plant biochemistry, and contribute to the growing set of resources for studying lepidopteran ecology and evolution.

## Genome sequence report

**Figure 1.**
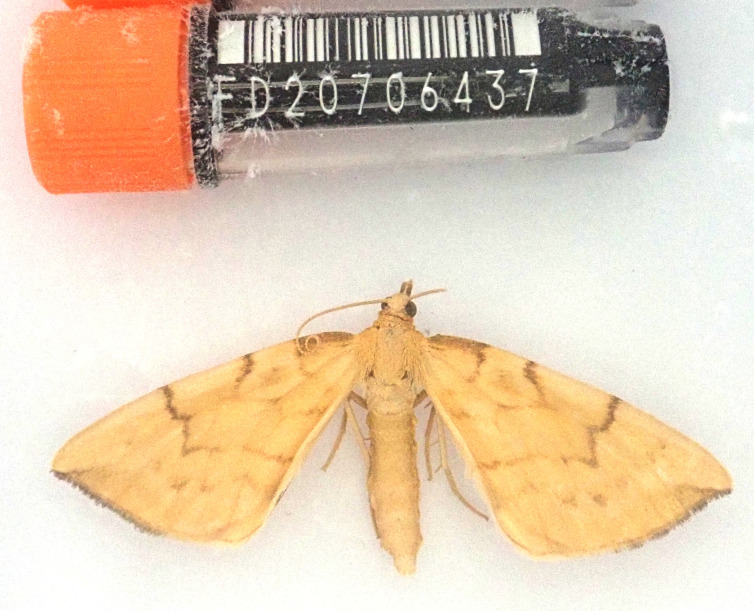
Photograph of the *Gandaritis pyraliata* (ilGanPyra1) specimen used for genome sequencing.

The genome was sequenced from one female *Gandaritis pyraliata* ( Figure 1) collected from Wytham Woods, Oxfordshire, UK (51.76, –1.34). A total of 31-fold coverage in Pacific Biosciences single-molecule HiFi long reads was generated. Primary assembly contigs were scaffolded with chromosome conformation Hi-C data. Manual assembly curation corrected 17 missing joins or mis-joins and removed 3 haplotypic duplications, reducing the scaffold number by 25.93%.

**Figure 2.**
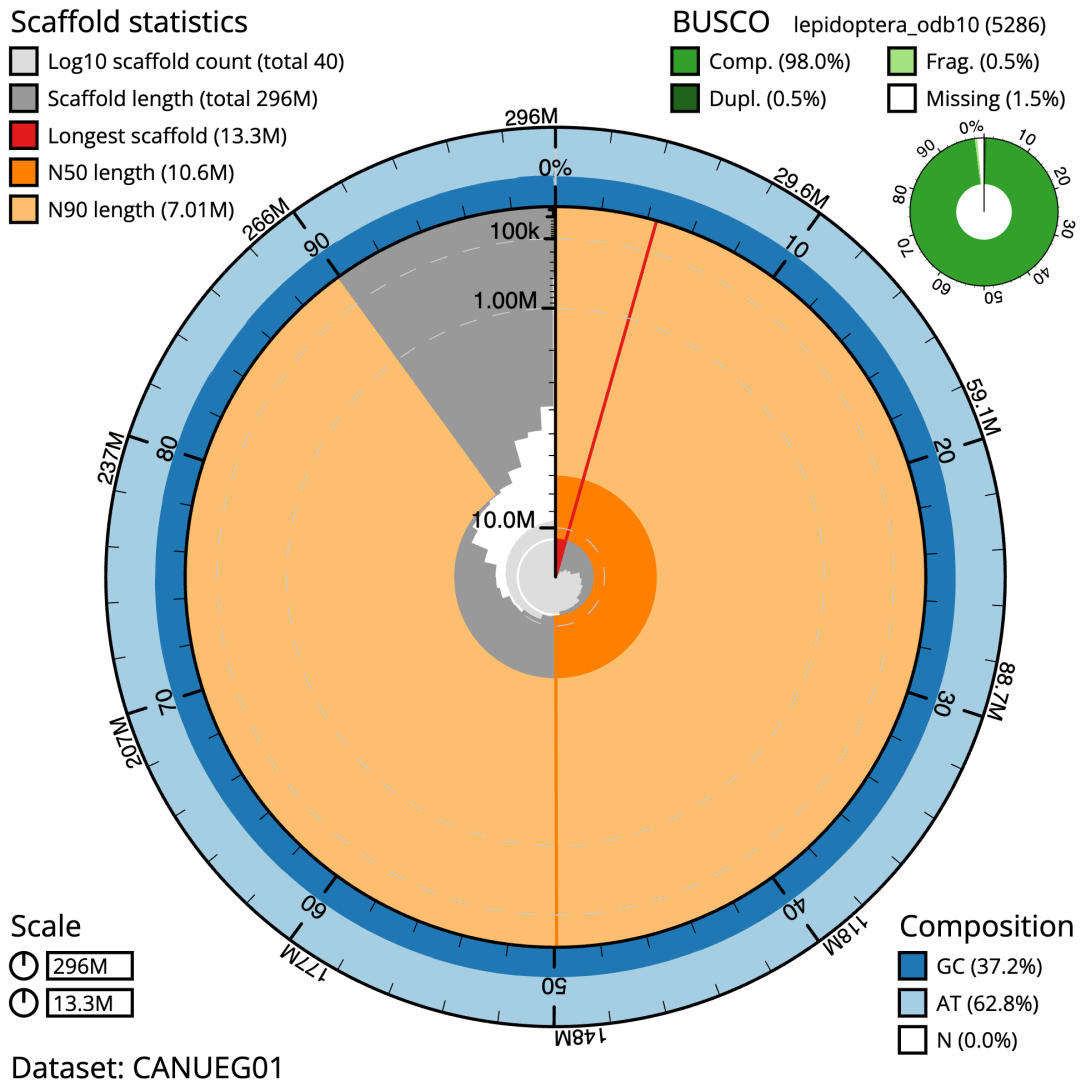
Genome assembly of *Gandaritis pyraliata*, ilGanPyra1.1: metrics. The BlobToolKit Snailplot shows N50 metrics and BUSCO gene completeness. The main plot is divided into 1,000 size-ordered bins around the circumference with each bin representing 0.1% of the 295,653,368 bp assembly. The distribution of scaffold lengths is shown in dark grey with the plot radius scaled to the longest scaffold present in the assembly (13,285,173 bp, shown in red). Orange and pale-orange arcs show the N50 and N90 scaffold lengths (10,572,331 and 7,009,674 bp), respectively. The pale grey spiral shows the cumulative scaffold count on a log scale with white scale lines showing successive orders of magnitude. The blue and pale-blue area around the outside of the plot shows the distribution of GC, AT and N percentages in the same bins as the inner plot. A summary of complete, fragmented, duplicated and missing BUSCO genes in the lepidoptera_odb10 set is shown in the top right. An interactive version of this figure is available at https://blobtoolkit.genomehubs.org/view/ilGanPyra1.1/dataset/CANUEG01/snail.

The final assembly has a total length of 295.6 Mb in 39 sequence scaffolds with a scaffold N50 of 10.6 Mb ( Table 1). Most (99.94%) of the assembly sequence was assigned to 31 chromosomal-level scaffolds, representing 29 autosomes and the W and Z sex chromosomes. Chromosome-scale scaffolds confirmed by the Hi-C data are named in order of size ( Figure 2– Figure 5; Table 2). While not fully phased, the assembly deposited is of one haplotype. Contigs corresponding to the second haplotype have also been deposited. The mitochondrial genome was also assembled and can be found as a contig within the multifasta file of the genome submission.

**Table 1.** Genome data for *Gandaritis pyraliata*, ilGanPyra1.1.

Project accession data		
Assembly identifier	ilGanPyra1.1	
Species	*Gandaritis pyraliata*	
Specimen	ilGanPyra1	
NCBI taxonomy ID	934938	
BioProject	PRJEB56798	
BioSample ID	SAMEA10978764	
Isolate information	ilGanPyra1, head and thorax (DNA sequencing and Hi-C scaffolding)	
Assembly metrics *		*Benchmark*
Consensus quality (QV)	65.5	*≥ 50*
*k*-mer completeness	100%	*≥ 95%*
BUSCO **	C:98.0%[S:97.4%,D:0.5%], F:0.5%,M:1.5%,n:5,286	*C ≥ 95%*
Percentage of assembly mapped to chromosomes	99.94%	*≥ 95%*
Sex chromosomes	W and X chromosomes	*localised homologous pairs*
Organelles	Mitochondrial genome assembled	*complete single alleles*
Raw data accessions		
PacificBiosciences SEQUEL II	ERR10395976	
Hi-C Illumina	ERR10395982	
Genome assembly		
Assembly accession	GCA_947859175.1	
*Accession of alternate haplotype*	GCA_947859165.1	
Span (Mb)	295.6	
Number of contigs	80	
Contig N50 length (Mb)	8.3	
Number of scaffolds	40	
Scaffold N50 length (Mb)	10.6	
Longest scaffold (Mb)	13.3	
Genome annotation		
Number of protein-coding genes	15,805	
Number of gene transcripts	15,998	

* Assembly metric benchmarks are adapted from column VGP-2020 of “Table 1: Proposed standards and metrics for defining genome assembly quality” from ( Rhie *et al.*, 2021). ** BUSCO scores based on the lepidoptera_odb10 BUSCO set using v5.3.2. C = complete [S = single copy, D = duplicated], F = fragmented, M = missing, n = number of orthologues in comparison. A full set of BUSCO scores is available at https://blobtoolkit.genomehubs.org/view/ilGanPyra1.1/dataset/CANUEG01/busco.

**Figure 3.**
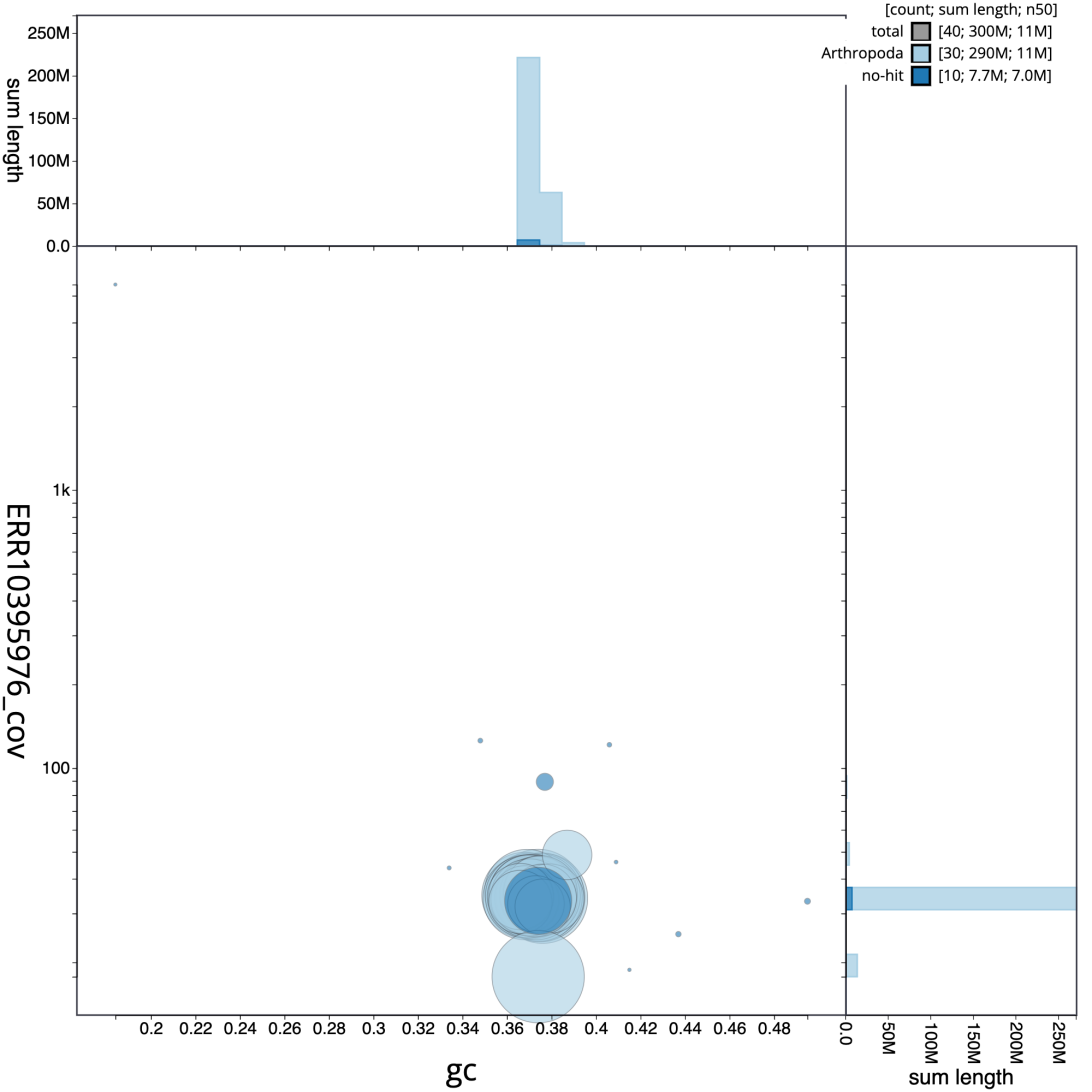
Genome assembly of *Gandaritis pyraliata*, ilGanPyra1.1: BlobToolKit GC-coverage plot. Scaffolds are coloured by phylum. Circles are sized in proportion to scaffold length. Histograms show the distribution of scaffold length sum along each axis. An interactive version of this figure is available at https://blobtoolkit.genomehubs.org/view/ilGanPyra1.1/dataset/CANUEG01/blob.

**Figure 4.**
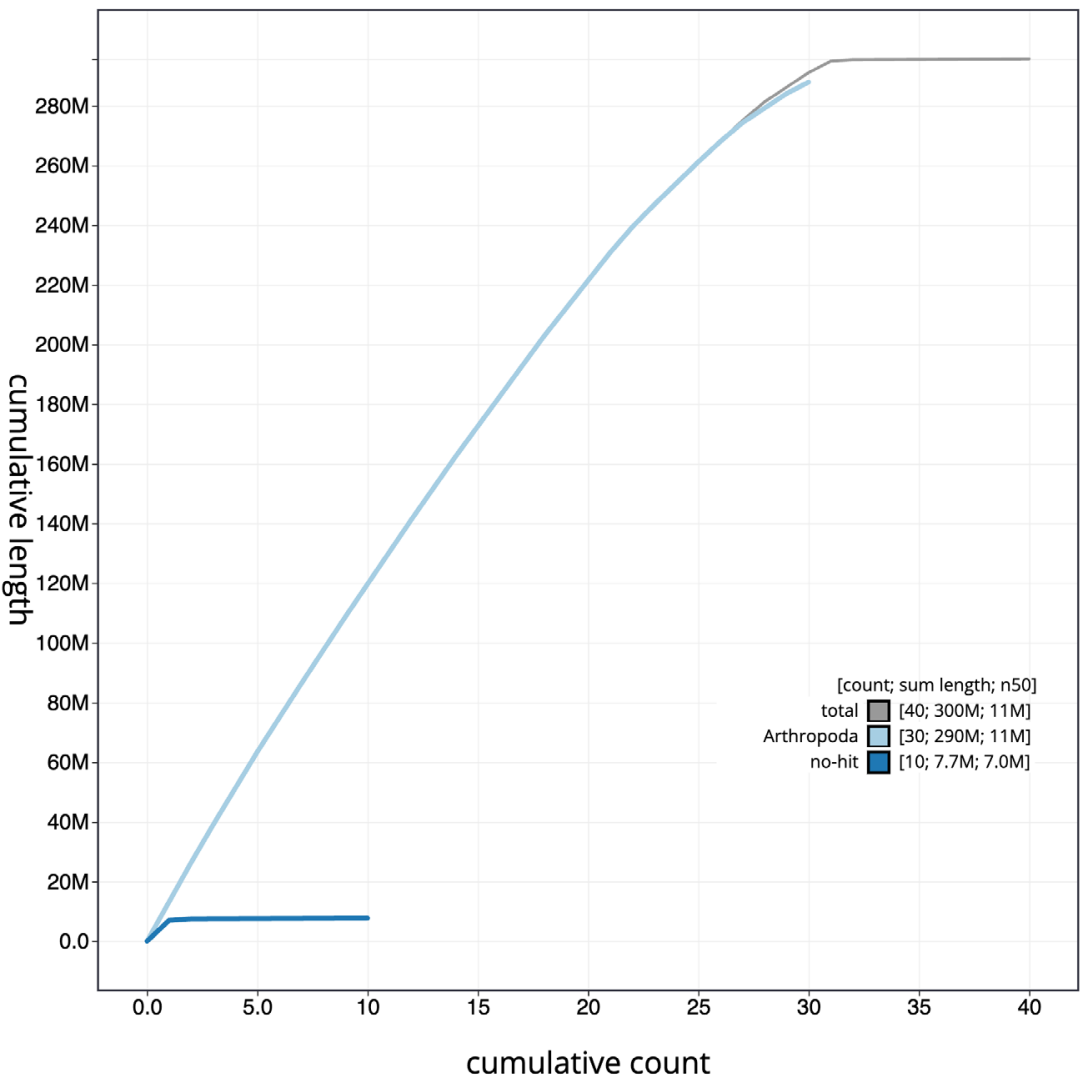
Genome assembly of *Gandaritis pyraliata*, ilGanPyra1.1: BlobToolKit cumulative sequence plot. The grey line shows cumulative length for all scaffolds. Coloured lines show cumulative lengths of scaffolds assigned to each phylum using the buscogenes taxrule. An interactive version of this figure is available at https://blobtoolkit.genomehubs.org/view/ilGanPyra1.1/dataset/CANUEG01/cumulative.

**Figure 5.**
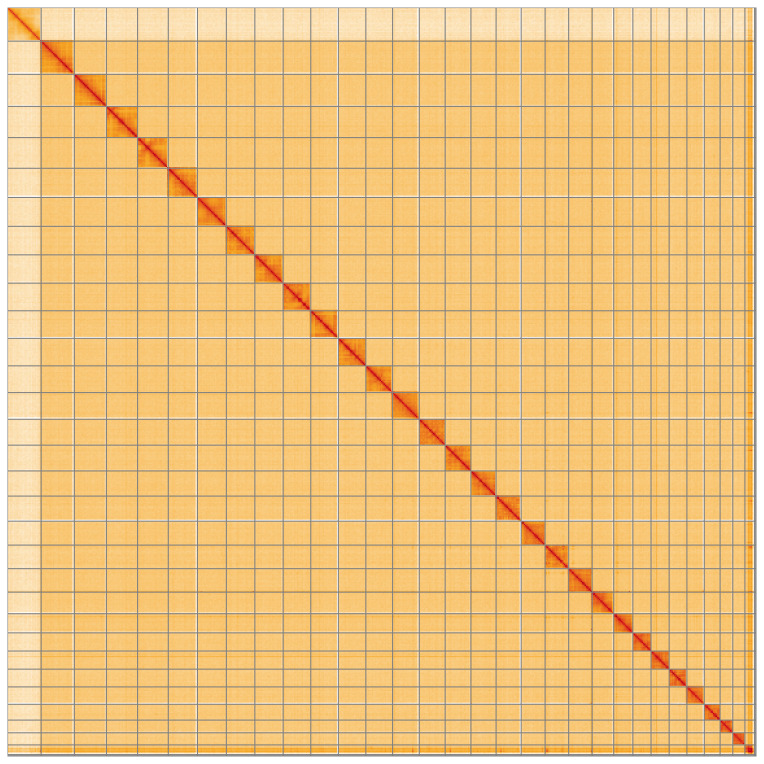
Genome assembly of *Gandaritis pyraliata*, ilGanPyra1.1: Hi-C contact map of the ilGanPyra1.1 assembly, visualised using HiGlass. Chromosomes are shown in order of size from left to right and top to bottom. An interactive version of this figure may be viewed at https://genome-note-higlass.tol.sanger.ac.uk/l/?d=DNbo17mrReWbHG50fYszFg.

**Table 2.** Chromosomal pseudomolecules in the genome assembly of *Gandaritis pyraliata*, ilGanPyra1.

INSDC accession	Chromosome	Length (Mb)	GC%
OX401851.1	1	13.1	37.5
OX401852.1	2	12.66	37.5
OX401853.1	3	12.31	37.5
OX401854.1	4	12.12	37.5
OX401855.1	5	11.53	37.0
OX401856.1	6	11.4	37.0
OX401857.1	7	11.25	37.0
OX401858.1	8	11.18	37.0
OX401859.1	9	10.91	37.0
OX401860.1	10	10.88	37.5
OX401861.1	11	10.85	37.0
OX401862.1	12	10.57	37.0
OX401863.1	13	10.53	37.5
OX401864.1	14	10.21	37.0
OX401865.1	15	10.18	37.0
OX401866.1	16	9.94	37.5
OX401867.1	17	9.89	37.5
OX401868.1	18	9.46	37.0
OX401869.1	19	9.3	37.5
OX401870.1	20	9.27	37.0
OX401871.1	21	8.53	37.5
OX401872.1	22	7.53	38.0
OX401873.1	23	7.19	37.0
OX401874.1	24	7.15	37.5
OX401875.1	25	7.01	37.5
OX401876.1	26	6.86	36.5
OX401877.1	27	6.26	36.5
OX401878.1	28	5.01	37.5
OX401879.1	29	4.78	37.5
OX401880.1	W	3.82	38.5
OX401850.1	Z	13.29	37.5
OX401881.1	MT	0.02	18.5

The estimated Quality Value (QV) of the final assembly is 65.5 with *k*-mer completeness of 100%, and the assembly has a BUSCO v5.3.2 completeness of 98.0% (single = 97.4%, duplicated = 0.5%), using the lepidoptera_odb10 reference set ( *n* = 5,286).

Metadata for specimens, spectral estimates, sequencing runs, contaminants and pre-curation assembly statistics can be found at https://links.tol.sanger.ac.uk/species/934938.

## Genome annotation report

The *Gandaritis pyraliata* genome assembly (GCA_947859175.1) was annotated using the Ensembl rapid annotation pipeline ( Table 1; https://rapid.ensembl.org/Gandaritis_pyraliata_GCA_947859175.1/Info/Index). The resulting annotation includes 15,998 transcribed mRNAs from 15,805 protein-coding genes. 

## Methods

### Sample acquisition and nucleic acid extraction

The specimen selected for genome sequencing was a female *Gandaritis pyraliata* (specimen number Ox001597, ilGanPyra1) collected from Wytham Woods, Oxfordshire (biological vice-county Berkshire), UK (latitude 51.76, longitude –1.34) on 2021-06-30. The specimen was taken from woodland habitat by Douglas Boyes (University of Oxford) using a light trap. The specimen was identified by the collector and snap-frozen on dry ice.

The ilGanPyra1 sample was prepared for DNA sequencing at the Tree of Life laboratory, Wellcome Sanger Institute (WSI). The tissue was weighed and dissected on dry ice with tissue set aside for Hi-C sequencing. Head and thorax tissue was disrupted using a Nippi Powermasher fitted with a BioMasher pestle. DNA was extracted at the Wellcome Sanger Institute (WSI) Scientific Operations core using the Qiagen MagAttract HMW DNA kit, according to the manufacturer’s instructions.

### Sequencing

Pacific Biosciences HiFi circular consensus DNA sequencing libraries were constructed according to the manufacturers’ instructions. DNA sequencing was performed by the Scientific Operations core at the WSI on Pacific Biosciences SEQUEL II (HiFi) instrument. Hi-C data were also generated from head and thorax tissue of ilGanPyra1 that had been set aside, using the Arimav2 kit and sequenced on the Illumina NovaSeq 6000 instrument.

### Genome assembly, curation and evaluation

Assembly was carried out with Hifiasm ( Cheng *et al.*, 2021) and haplotypic duplication was identified and removed with purge_dups ( Guan *et al.*, 2020). The assembly was then scaffolded with Hi-C data ( Rao *et al.*, 2014) using YaHS ( Zhou *et al.*, 2023). The assembly was checked for contamination and corrected as described previously ( Howe *et al.*, 2021). Manual curation was performed using HiGlass ( Kerpedjiev *et al.*, 2018) and Pretext ( Harry, 2022). The mitochondrial genome was assembled using MitoHiFi ( Uliano-Silva *et al.*, 2022), which runs MitoFinder ( Allio *et al.*, 2020) or MITOS ( Bernt *et al.*, 2013) and uses these annotations to select the final mitochondrial contig and to ensure the general quality of the sequence.

A Hi-C map for the final assembly was produced using bwa-mem2 ( Vasimuddin *et al.*, 2019) in the Cooler file format ( Abdennur & Mirny, 2020). To assess the assembly metrics, the *k*-mer completeness and QV consensus quality values were calculated in Merqury ( Rhie *et al.*, 2020). This work was done using Nextflow ( Di Tommaso *et al.*, 2017) DSL2 pipelines “sanger-tol/readmapping” ( Surana *et al.,* 2023a) and “sanger-tol/genomenote” ( Surana *et al.*, 2023b). The genome was analysed within the BlobToolKit environment ( Challis *et al.*, 2020) and BUSCO scores ( Manni *et al.*, 2021; Simão *et al.*, 2015) were calculated.

Table 3 contains a list of relevant software tool versions and sources.

**Table 3.** Software tools: versions and sources.

Software tool	Version	Source
BlobToolKit	4.1.5	https://github.com/blobtoolkit/ blobtoolkit
BUSCO	5.3.2	https://gitlab.com/ezlab/busco
Hifiasm	0.16.1-r375	https://github.com/chhylp123/ hifiasm
HiGlass	1.11.6	https://github.com/higlass/higlass
Merqury	MerquryFK	https://github.com/ thegenemyers/MERQURY.FK
MitoHiFi	2	https://github.com/ marcelauliano/MitoHiFi
PretextView	0.2	https://github.com/wtsi-hpag/ PretextView
purge_dups	1.2.3	https://github.com/dfguan/ purge_dups
sanger-tol/ genomenote	v1.0	https://github.com/sanger-tol/ genomenote
sanger-tol/ readmapping	1.1.0	https://github.com/sanger-tol/ readmapping/tree/1.1.0
YaHS	yahs- 1.1.91eebc2	https://github.com/c-zhou/yahs

### Genome annotation

The BRAKER2 pipeline ( Brůna *et al.*, 2021) was used in the default protein mode to generate annotation for the *Gandaritis pyraliata* assembly (GCA_947859175.1) in Ensembl Rapid Release.

### Wellcome Sanger Institute - Legal and Governance

The materials that have contributed to this genome note have been supplied by a Darwin Tree of Life Partner. The submission of materials by a Darwin Tree of Life Partner is subject to the **‘Darwin Tree of Life Project Sampling Code of Practice’**, which can be found in full on the Darwin Tree of Life website here. By agreeing with and signing up to the Sampling Code of Practice, the Darwin Tree of Life Partner agrees they will meet the legal and ethical requirements and standards set out within this document in respect of all samples acquired for, and supplied to, the Darwin Tree of Life Project.

Further, the Wellcome Sanger Institute employs a process whereby due diligence is carried out proportionate to the nature of the materials themselves, and the circumstances under which they have been/are to be collected and provided for use. The purpose of this is to address and mitigate any potential legal and/or ethical implications of receipt and use of the materials as part of the research project, and to ensure that in doing so we align with best practice wherever possible. The overarching areas of consideration are:

- Ethical review of provenance and sourcing of the material
- Legality of collection, transfer and use (national and international)

Each transfer of samples is further undertaken according to a Research Collaboration Agreement or Material Transfer Agreement entered into by the Darwin Tree of Life Partner, Genome Research Limited (operating as the Wellcome Sanger Institute), and in some circumstances other Darwin Tree of Life collaborators.

## Data Availability

European Nucleotide Archive: *Gandaritis pyraliata* (barred straw). Accession number PRJEB56798; https://identifiers.org/ena.embl/PRJEB56798. ( Wellcome Sanger Institute, 2023) The genome sequence is released openly for reuse. The *Gandaritis pyraliata* genome sequencing initiative is part of the Darwin Tree of Life (DToL) project. All raw sequence data and the assembly have been deposited in INSDC databases. Raw data and assembly accession identifiers are reported in Table 1.

## Author information

Members of the University of Oxford and Wytham Woods Genome Acquisition Lab are listed here: https://doi.org/10.5281/zenodo.4789928.

Members of the Darwin Tree of Life Barcoding collective are listed here: https://doi.org/10.5281/zenodo.4893703.

Members of the Wellcome Sanger Institute Tree of Life programme are listed here: https://doi.org/10.5281/zenodo.4783585.

Members of Wellcome Sanger Institute Scientific Operations: DNA Pipelines collective are listed here: https://doi.org/10.5281/zenodo.4790455.

Members of the Tree of Life Core Informatics collective are listed here: https://doi.org/10.5281/zenodo.5013541.

Members of the Darwin Tree of Life Consortium are listed here: https://doi.org/10.5281/zenodo.4783558.
