# Supplementary material for: The genome sequence of the Barred Straw, Gandaritis pyraliata (Denis & Schiffermüller, 1775)
Source: Wellcome Open Res. 2025 Oct 15;8:435. Originally published 2023 Oct 10. [Version 2] doi: 10.12688/wellcomeopenres.19526.2 (PMC12811725; doi:10.12688/wellcomeopenres.19526.2)
Supplement: Peer review history

# Reviewer report: Reviewer response for version 2

**DOI:** 10.21956/wellcomeopenres.27604.r145061

**Published:** 2026-01-28

**Authors:** Olli-Pekka Smolander

**Affiliations:** 1 University of Helsinki, Helsinki, Finland

The authors describe chromosomal-level genome assembly of the Barred Straw moth, *Gandaritis pyraliata*. Pacific Biosciences HiFi long-read sequencing and Illumina sequenced Hi-C data are used for contig assembly and scaffolding, respectively. The authors report that the assembly spans 295.6 Mb, with 99.94% of the sequence localized to 31 chromosomal pseudomolecules, including 29 autosomes and the W and Z sex chromosomes. Annotation was conducted using the Ensembl Rapid Release pipeline (with BRAKER2 in protein mode), identifying 15,805 protein-coding genes. BUSCO analysis against the lepidoptera_odb10 set shows high completeness at 98.0%. The result is a solid genomic resource for investigating the evolutionary biochemistry of this species and its specialised interactions with chemically unfavourable host plants.

 The rationale and context (ecology and evolutionary adaptation of the species) for creating this dataset is clearly described. Furthermore, the datasets are presented in a usable and accessible format. The raw data and assembly have been deposited in appropriate international databases, and the authors provide accession numbers to ensure public access.

 However, the protocols and technical soundness are only partially sufficient. While there are no questions about the quality of the assembly and the used methods, the lack of transcriptomic evidence is a significant factor affecting the annotation quality. The use BRAKER2 in default protein mode without species-specific RNA-Seq data means that the structural accuracy of gene models, particularly intron/exon boundaries, is uncertain. Furthermore, the methodological details for separating the W and Z sex chromosomes from autosomes is not described at an adequate level. To achieve complete technical clarity, it should be described if this identification was based for example on coverage analysis, homology to other *Geometridae* genomes, or specific Hi-C features. Additionally, it would be good to list all the software tools (e.g. BRAKER2 and Cooler) that have been part of this work. Similarly, for each tool, it should be stated whether default parameters were used and if not, how those were modified.

Are sufficient details of methods and materials provided to allow replication by others?

Partly

Is the rationale for creating the dataset(s) clearly described?

Yes

Are the datasets clearly presented in a useable and accessible format?

Yes

Are the protocols appropriate and is the work technically sound?

Partly

Reviewer Expertise:

Genomics, computational biology, bioinformatics, microbiome, ML

I confirm that I have read this submission and believe that I have an appropriate level of expertise to confirm that it is of an acceptable scientific standard, however I have significant reservations, as outlined above.

# Reviewer report: Reviewer response for version 2

**DOI:** 10.21956/wellcomeopenres.27604.r144592

**Published:** 2026-01-16

**Authors:** Yu-Feng Huang

**Affiliations:** 1 Computer Science and Engineering, Yuan Ze University (Ringgold ID: 34895), Zhongli District, Taoyuan City, Taiwan

This revised Data Note reports a high-quality reference genome assembly for *Gandaritis pyraliata*, generated using PacBio HiFi and Illumina Hi-C sequencing data. The assembly metrics are robust, achieving a combined Consensus Quality (QV) of 66.9 and a Scaffold N50 of 10.6 Mb. The authors successfully assigned 99.94% of the sequence to 31 chromosomal-level scaffolds, a result that meets and exceeds Earth BioGenome Project standards. However, although the manuscript has been revised to address prior peer review comments, inconsistencies regarding scaffold counts persist between the text and figures. Furthermore, specific biological validations namely gene content verification, mitogenome circularity, and sex chromosome identification - require additional clarification to ensure the data's reliability for downstream research.

**Major Comments**

**1. Gene Annotation Validation **The manuscript now includes a genome annotation generated via the BRAKER2 pipeline in "default protein mode," which identified 15,805 protein-coding genes. This annotation appears to be derived solely from protein homology, as there is no mention of species-specific RNA-Seq data generation in the Sample Acquisition or Methods sections. Consequently, the structural accuracy of these gene models, particularly intron/exon boundaries, remains unverified against transcriptomic evidence. Please explicitly state the limitations of this annotation method within the text. If feasible, please perform a spot-check of a well-conserved gene family to confirm that the protein-mode prediction has yielded biologically valid gene structures; alternatively, clarify that this dataset serves as a preliminary set of gene models pending transcriptomic validation.

**2. Sex Chromosome Identification Methodology **The assembly assigns scaffolds to "29 autosomes and the W and Z sex chromosomes". However, the manuscript lacks a methodological description explaining how the W and Z chromosomes were identified and distinguished from the autosomes. Please specify the evidentiary basis for this assignment. For instance, was the identification based on coverage analysis (e.g., observing half-coverage for the Z/W chromosomes in the female specimen), homology to other *Geometridae* genomes, or specific Hi-C contact patterns?

**Minor Comments**

**3. Mitogenome Circularization Verification** The mitochondrial genome was assembled using MitoHiFi. The mitochondrial genome size reported in the text differs from the value listed in the Table 2. While the text notes that MitoHiFi ensures "general quality," it does not explicitly confirm that the assembly was verified as circular. Please confirm whether the circular topology was validated (e.g., by checking for overlapping ends) and if the sequence was rotated to a standard start feature to ensure it represents the complete organelle genome.

**4. Software Tools (Table 3)** Table 3 lists the software versions utilized in this study. However, discrepancies exist between the methods text and the table. Specifically, the BRAKER2 pipeline used for annotation is not listed. Additionally, Cooler, which is mentioned in the methods regarding Hi-C map production, is absent from the table. Please audit Table 3 and update it to include these missing tools along with their respective version numbers.

Are sufficient details of methods and materials provided to allow replication by others?

Yes

Is the rationale for creating the dataset(s) clearly described?

Yes

Are the datasets clearly presented in a useable and accessible format?

Yes

Are the protocols appropriate and is the work technically sound?

Partly

Reviewer Expertise:

Bioinformatics, Cancer Bioinformatics, Mitogenomics, Genome sequencing, assembly and gene annotation.

I confirm that I have read this submission and believe that I have an appropriate level of expertise to confirm that it is of an acceptable scientific standard, however I have significant reservations, as outlined above.

# Reviewer report: Reviewer response for version 2

**DOI:** 10.21956/wellcomeopenres.27604.r136391

**Published:** 2025-10-25

**Authors:** Alessandro Grapputo

**Affiliations:** 1 Universita degli Studi di Padova, Padua, Veneto, Italy

The revised paper by Boyes and collaborator have addressed appropriately the concerns I have raised in the previous review report. The paper can be indexed in the present form.

Are sufficient details of methods and materials provided to allow replication by others?

Partly

Is the rationale for creating the dataset(s) clearly described?

Yes

Are the datasets clearly presented in a useable and accessible format?

Yes

Are the protocols appropriate and is the work technically sound?

Yes

Reviewer Expertise:

Population genetics and genomics

I confirm that I have read this submission and believe that I have an appropriate level of expertise to confirm that it is of an acceptable scientific standard.

# Reviewer report: Reviewer response for version 1

**DOI:** 10.21956/wellcomeopenres.21629.r83549

**Published:** 2024-05-28

**Authors:** Fahad Alqahtani

**Affiliations:** 1 King Abdulaziz City for Science and Technology, Riyadh, Saudi Arabia

The authors of "The genome sequence of the Barred Straw, Gandaritis pyraliata (Denis & Schiffermüller, 1775)," have achieved a highly complete genome assembly at the chromosome level for a female Gandaritis pyraliata. They reconstructed the genome assembly using two sequencing technologies: Pacific Biosciences SEQUEL II and Hi-C Illumina. BUSCO analysis was used to assess the completeness of the genome assembly, indicating a genome size of 295.6 megabases, with 98% of common genes in the lepidoptera_odb10 present.

 There are a few minor comments that should be addressed:

- The number of contigs in Table 1 is 80, while it is reported in NCBI as 79.
- The number of scaffolds in Table 1 is 40, while it is reported in NCBI as 39.
- 'Pretext' in the Genome Assembly section should be corrected to 'PretextView' to match the name in Table 3.

Are sufficient details of methods and materials provided to allow replication by others?

Yes

Is the rationale for creating the dataset(s) clearly described?

Yes

Are the datasets clearly presented in a useable and accessible format?

Yes

Are the protocols appropriate and is the work technically sound?

Yes

Reviewer Expertise:

Bioinformatics

I confirm that I have read this submission and believe that I have an appropriate level of expertise to confirm that it is of an acceptable scientific standard, however I have significant reservations, as outlined above.

# Reviewer report: Reviewer response for version 1

**DOI:** 10.21956/wellcomeopenres.21629.r80784

**Published:** 2024-05-16

**Authors:** Alessandro Grapputo

**Affiliations:** 1 Universita degli Studi di Padova, Padua, Veneto, Italy

The paper by Boyes and colleagues present a new genome in the tree of life project, the genome sequence and annotation of the Barred Straw, *Gandaritis pyraliata*, a lepidoptera of the family Geometridae. The Barred Straw (synonym *Eulithis* *pyraliata*), as the other close relatives *Eulithis* species, *E. mellinata* and *E. populate*, usually rests in a very distinctive and characteristic way with the forewings held out at 90° to the body and with the hindwings hidden behind them.

 The methods for sequencing acquisition, the genome assembly pipeline and annotation with the Ensembl rapid annotation pipeline follow the Classique scheme of most Data Note papers published by Wellcome Open Research. The methods are sound and  good and I do not have comments about that. However the software listed have many optional parameters and none have been reported. Therefore to the question: Are sufficient details of methods and materials provided to allow replication by others? I would say: partly. If the default parameters have been used this should be stated for each software.

 The paper is well written, however the first paragraph of the background would benefit, first of at least a reference for all the information about the species reported, and second from adding the comparison of the characteristic way of resting with other members of the genus (possibly it had evolved only once and the phylogenomic analysis of the group may shed some light on it).

Are sufficient details of methods and materials provided to allow replication by others?

Partly

Is the rationale for creating the dataset(s) clearly described?

Yes

Are the datasets clearly presented in a useable and accessible format?

Yes

Are the protocols appropriate and is the work technically sound?

Yes

Reviewer Expertise:

Population genetics and genomics

I confirm that I have read this submission and believe that I have an appropriate level of expertise to confirm that it is of an acceptable scientific standard, however I have significant reservations, as outlined above.
